# Early reduction in cardiorespiratory fitness and diastolic reserve following radiation therapy for lung cancer

**DOI:** 10.1186/s40959-024-00216-2

**Published:** 2024-03-12

**Authors:** Georgia Thomas, Elisabeth Weiss, Marco Giuseppe Del Buono, Francesco Moroni, Josh West, Rachel Myers, Emily Kontos, Michele Golino, Antonio Abbate, Justin M. Canada

**Affiliations:** 1 VCU Pauley Heart Center, Virginia Commonwealth University, 1200 East Broad Street, Richmond, VA 23298 PO Box 980335, USA; 2 VCU Massey Cancer Center; Department of Radiation Oncology, Virginia Commonwealth University, Richmond, VA USA; 3 Berne Cardiovascular Research Center and Department of Medicine, University of Virginia, Charlottesville, VA USA; 4 Department of Medicine and Surgery, University of Insubria, Varese, Italy

**Keywords:** Diastolic reserve, Radiotherapy, Cardiorespiratory fitness, Lung cancer

## Abstract

**Background:**

Contemporary radiotherapy for the treatment of lung cancer is effective in targeting tumor tissue while limiting heart exposure, yet cardiac toxicity still occurs, often becoming clinically apparent years later. Cardiorespiratory fitness (CRF) is an independent predictor of cardiovascular, cancer-related, and overall mortality and may serve as a sensitive measure of subclinical cardiac toxicity following anti-cancer treatments. Prior work has demonstrated a significant relationship between reduced CRF and impaired left-ventricular (LV) diastolic reserve in cancer survivors following thoracic radiotherapy. The purpose of this study was to assess early longitudinal changes in CRF and cardiac function in patients with lung cancer following radiotherapy.

**Methods:**

Ten patients (69 [61–76] years, 70% female) with lung cancer without known cardiovascular disease scheduled to receive radiotherapy involving a clinically-relevant heart dose (≥ 5 Gy to > 10% of heart volume) were evaluated prior to and following treatment. Changes in CRF (peak oxygen consumption [VO_2peak_], oxygen uptake efficiency slope [OUES]), cardiac function (LV ejection fraction [LVEF], rest and exercise diastolic function [diastolic functional reserve index (DFRI)]), cardiac biomarkers (N-terminal pro-brain natriuretic peptide [NT-proBNP], high-sensitivity C-reactive protein [hsCRP]), and health-related quality of life (HRQOL; Functional Assessment of Cancer Therapy-General-7 [FACT-G7]) were measured.

**Results:**

The VO_2peak_ was reduced at baseline (1.245 [0.882–1.605] L·min^− 1^; 70 [62–86] %-predicted) and significantly declined (1.095 [0.810–1.448] L·min^− 1^, *P* = 0.047; 62 [56–76] %-predicted, *P* = 0.005) at 6.0 [3.0–6.0] months post-radiotherapy. Similarly, a significant decline in the OUES was observed (1.63 [1.27–1.88] to 1.57 [1.12–1.75], *P* = 0.032). Systolic cardiac function was normal at baseline and did not change following radiotherapy (LVEF; 62 [56–65]% to 66 [57–68]%, *P* = 0.475). The DFRI significantly declined following radiotherapy (34.9 [22.7–41.6] vs. 12.8 [3.1–35.9]). The hsCRP increased significantly from 4.4 [1.4–5.8] to 6.1 [3.7–20.7] g/L, *P* = 0.047 with a trend towards higher levels of NT-proBNP (65 [49–125] to 121 [88–191] pg/mL, *P* = 0.110). Health-related quality of life significantly decreased (FACT-G7; 21.5 [18.8–25] to 15.5 [11.5–20]; *P* = 0.021) post-radiotherapy.

**Conclusions:**

Patients with lung cancer receiving radiotherapy with a clinically-significant heart dose experience reductions in CRF (VO_2peak_, OUES) as early as six months following treatment with concurrent reductions in diastolic reserve (DFRI), HRQOL, and increases in cardiac biomarkers (NT-proBNP, hsCRP).

## Introduction

Radiotherapy is a standard treatment in patients with lung cancer. While it improves survival, it is associated with an increased risk of cardiovascular disease and is a leading cause of nonmalignant morbidity and mortality [1, 2]. Radiation-induced cardiac disease (RICD) is typically thought to be a late-occurring event, but studies have shown that early subclinical changes occur [2, 3]. Patients with lung cancer are at high cardiovascular risk at baseline (i.e., due to tobacco smoking history, increasing prevalence of shared cardiovascular disease (CVD) risk factors, advancing age, and comorbid chronic obstructive pulmonary disease [COPD]) which likely shortens the latency period between radiation exposure and subsequent cardiotoxicity. Commonly used tools to assess cardiac function (i.e., left ventricular ejection fraction [LVEF] with echocardiography) are known to be insensitive to minor injury and therefore subtle changes in myocardial systolic or diastolic function may go unnoticed for many years [4]. Indeed, radiation-induced cardiomyopathy typically presents more frequently with LV diastolic function abnormalities antecedent to declines in systolic function [3, 5, 6]. 

There is a growing body of literature that underscores the assessment of cardiorespiratory fitness (CRF) to stratify risk in the cancer patient [7–10] suggesting it may serve as an integrated functional biomarker to detect cardiotoxicity [11–13]. Cardiopulmonary exercise testing (CPET) is the gold standard for evaluating integrative cardiovascular function and yields an objective quantifiable measure of CRF through the measurement of peak oxygen consumption (VO_2peak_) and the oxygen uptake efficiency slope (OUES), surrogates for quality of life and survival in patients with lung cancer and heart failure [10, 14–17]. Previous work has demonstrated significant CRF impairment and a strong inverse relationship between CRF and survival in patients with lung cancer [18–20] with the mechanisms of impairment being likely multifactorial due to multiple derangements in the oxygen cascade (i.e., impairments in respiratory, cardiovascular, and musculoskeletal function).

Our previous work has demonstrated an independent association between VO_2peak_ and the diastolic functional reserve index (DFRI), a Doppler-stress echocardiography measurement accounting for resting and exercise-induced changes in early diastolic mitral annular velocity (e’), in patients who were free of overt cardiovascular disease and had previously received chest radiotherapy, where a decreased DFRI was associated with reduced CRF [21]. Additionally, metrics of CRF were inversely associated with LV extracellular volume fraction [22], a marker of diffuse myocardial fibrosis known to play a role in the pathophysiology of RICD [23]. Furthermore, in patients receiving radiotherapy for lung cancer with reduced respiratory function, we’ve shown that diastolic dysfunction contributes to reduced CRF, and that serum levels of N-terminal pro-brain natriuretic peptide (NT-proBNP) independently predicted VO_2peak_ [24]. These data suggest that changes in CRF and associated changes in cardiac diastolic reserve may serve as novel markers of RICD. The purpose of this study was to assess early longitudinal changes in CRF (VO_2peak_, OUES) and cardiac function with a focus on exercise diastolic reserve in adults with lung cancer following radiotherapy with an incidental heart dose. We hypothesized that individuals with lung cancer receiving radiotherapy would experience interval declines in CRF and cardiac diastolic reserve.

## Methods

The primary aim of this pilot study was to study longitudinal changes in CRF (measured as VO_2peak_ and the OUES) in adult patients with lung cancer undergoing radiotherapy treatment with an incidental heart dose ≥ 5 Gy (Gy) to > 10% heart volume who were able to undergo symptom-limited treadmill exercise testing. Secondary aims were to additionally study changes in cardiac diastolic reserve, blood biomarkers (high-sensitivity C-reactive protein [hsCRP], NT-proBNP), health-related quality of life, and metrics influencing the exercise response (respiratory function, physical activity levels, body habitus).

This study included patients (≥ 21 years of age) with locally advanced lung cancer within the Virginia Commonwealth University Massey Cancer Center Radiation Oncology clinics who were scheduled to receive radiotherapy with incidental heart exposure of ≥ 5 Gy to > 10% of the heart volume with no or minimal radiation dose to the heart previously (< 2 Gy mean heart dose). Exclusion criteria was contraindications to exercise testing as defined by the American Heart Association [25]. The study was approved by the local Institutional Review Board (HM20017432) and all subjects provided informed consent before enrollment. Analysis was limited to patients who completed both baseline and post-radiotherapy follow-up visits. Radiation dose was calculated based on a volumetric computed tomography data set obtained during a treatment planning session. A radiation oncologist quantified the total and heart radiation doses including %-volumes (V) of the heart receiving ≥ 5, 10, 20, 30, 40, and 50 Gy, respectively. Presence of cardiovascular disease (CVD) was defined as prior history or diagnosis of heart failure, coronary artery, cerebrovascular, or peripheral artery disease or aortic atherosclerosis. The presence of traditional CVD risk factors (smoking, hyperlipidemia, hypertension, diabetes, sedentary lifestyle, and obesity), CVD medication use (beta-blockers, angiotensin converting enzyme inhibitors/angiotensin receptor blockers, statins), comorbidities (history of COPD, prior cancers, renal disease), and cancer type, stage, and treatments were collected from medical records review and patient interview.

Patients underwent symptom-limited treadmill CPET according to established guidelines using a low-level (∼ 2 mL·kg^− 1^·min^− 1^ (estimated VO_2_)/ 30-seconds) ramping protocol coupled with Doppler-stress echocardiography before and after radiotherapy [25, 26]. Cardiorespiratory fitness was measured as VO_2peak_ (highest average value in the final 30-seconds of exercise) and expressed in absolute (L·min^− 1^), relative (mL·kg^− 1^·min^− 1^), and %-predicted values using the reference values proposed by Wasserman and colleagues [27]. Impaired VO_2peak_ was defined as < 85% of predicted values [28]. Functional disability, a threshold for the ability to independently perform activities of daily living, was defined as a VO_2peak_ ≤ 18.0 mL·kg^− 1^·min^− 1^ [29]. The OUES, an effort-independent marker of CRF that strongly correlates with VO_2peak,_ [30] was calculated as the quotient of VO_2_/log-transformed minute ventilation (VE) throughout the entire exercise period. The OUES was selected as an additional CRF measurement in this population based on its ability to assess CRF in the instance of a submaximal effort using conventional maximal test criteria [31]. The respiratory exchange ratio (RER) was calculated as the quotient of carbon dioxide production (VCO_2_) divided by VO_2_ at peak exercise. The VE/VCO_2_ slope was calculated from the entire exercise period. Two-dimensional transthoracic echocardiography was performed at rest and immediately post-exercise to measure cardiac structure and function according to standard recommendations [32] with focus on Doppler-derived diastolic function (early [E]/late [A] transmitral velocities, lateral and septal e’, calculation of E/e’, and DFRI [rest e’ x Δstress e’]).

Anthropometrics (body mass index [BMI]), physical activity levels, pulmonary function testing, and blood biomarkers (hsCRP, NT-proBNP, hemoglobin) were obtained pre-exercise at baseline and follow-up visits. Elevated hsCRP was defined as ≥ 1 mg/L and elevated NT-proBNP was defined as ≥ 125 pg/mL, respectively [33, 34]. Physical activity levels were assessed using the International Physical Activity Questionnaire (IPAQ), a validated questionnaire [35]. Pulmonary function testing was performed according to standard recommendations and included measurements of forced vital capacity (FVC), forced expiratory volume-1 s (FEV-1), and diffusing capacity of lung for carbon monoxide (DLCO) corrected for hemoglobin levels [36, 37]. Health-related quality of life (HRQOL) was assessed using the Functional Assessment of Cancer Therapy-General-7 (FACT-G7) instrument [38]. 

Continuous data are reported as median [interquartile range] based on non-normal distribution of data using tests of normality (Shapiro-Wilk) prior to data analysis or number (%) for nominal variables. All analyses were performed with non-parametric tests due to the assumption of non-normally distributed data. Spearman’s rank correlation coefficients were used to assess bivariate relationships for continuous variables. Pre/post related-samples comparisons of cardiopulmonary variables were made using the Wilcoxon signed-rank test. Post-hoc comparisons of significant pre/post related-samples testing were performed on baseline clinical characteristics (Yes/No; CVD risk factors, CV medication use, comorbidities, cancer stage, durvalumab immunotherapy use) using the Mann-Whitney U test. Statistical analysis was performed using SPSS v29.0 (IBM Corp, Armonk, NY) with a *P*-value < 0.05 considered significant. A formal power calculation was not completed due to the exploratory nature of the study and the lack of available data on this population to inform the calculation.

## Results

Ten patients (70% White females, 80% Stage III-IV lung cancer, 69 [61–76] years of age, all Eastern Cooperative Oncology Group status 0–1) underwent assessments at 1.5 [1.0-2.5] months following lung cancer diagnosis and 6.5 [4.5–12.3] days prior to the start of radiotherapy. Post-radiotherapy assessments occurred at 7.0 [4.8–7.3] months following baseline and 6 [3.0–6.0] months following the completion of radiotherapy. None of the patients had established cardiovascular disease. Table 1 details the baseline CVD factors, cardiovascular medication use, and oncologic characteristics of the cohort. There was a high prevalence of CVD risk factors, CV medication use, and comorbid COPD at baseline. Additionally, three patients (30%) had a history of prior chest surgery of various complexity (video-assisted thoracic surgery [VATS] – no intervention; right-upper lobe lobectomy; VATS with wedge resection only). However, none of these were associated with significant interval differences (i.e., before versus after radiotherapy assessments) in CRF or echocardiogram parameters (all *P*-values > 0.05). All patients received concurrent chemotherapy (90% carboplatin/paclitaxel regimens). Sixty-percent also received durvalumab immunotherapy which was not associated with interval changes in cardiopulmonary variables (all *P*’s > 0.393). Total prescribed radiotherapy dose was 60 Gy delivered in 30 fractions (2 Gy/fraction), mean lung dose was 11.0 [8.8–13.7] Gy, mean heart dose was 8.1 [4.8–12.4] Gy, and volume of heart receiving 5 Gy was 39.0 [18.3–54.5] %. The %-volumes of the heart receiving V10, V20, V30, V40, and V50 Gy, are listed in Table 1. All patients were treated with volumetric modulated arc therapy (VMAT) using respiratory management and image guidance (IGRT) on linear accelerators (Truebeam, Varian Medical Systems, Palo Alto, CA, USA) following 4DCT-based treatment planning (Brilliance Big Bore, Philips Medical Systems, The Netherlands).

**Table 1:** Baseline Characteristics of the Cohort

Variable	Baseline
**Cardiovascular Disease Risk Factors**	
Hypertension	6 (60%)
Diabetes Mellitus	4 (40%)
Hyperlipidemia	6 (60%)
Tobacco smoking history	8 (80%)
Obesity	5 (50%)
Sedentary Lifestyle	4 (40%)
**Cardiovascular Medications**	
Beta-blockers	5 (50%)
ACE-I/ARB	5 (50%)
Statins	5 (50%)
**Comorbidities**	
COPD	5 (50%)
COPD stage (GOLD criteria)	Moderate: 4 (40%) Severe: 1 (10%)
Prior other cancers	4 (40%)
Chronic kidney disease	2 (20%)
**Oncologic Staging and Treatment**	
Lung Cancer Stage	IIB: 2 (20%) IIIA: 2 (20%) IIIB: 5 (50%) IV: 1 (10%)
Lung Cancer tissue type	Adenocarcinoma: 4 (40%) Squamous cell cancer: 3 (30%) Small cell cancer: 2 (20%) Poorly differentiated cancer: 1 (10%)
Tumor Location	
Left Upper Lobe Right Upper Lobe	6 (60%) 4 (40%)
Radiotherapy Parameters	
MLRD, Gy MCRD, Gy Heart Max Dose, Gy Heart V5 Gy, % Heart V10 Gy, % Heart V20 Gy, % Heart V30 Gy, % Heart V40 Gy, % Heart V50 Gy, %	11.0 [8.8–13.7] 8.1 [4.8–12.4] 65.3 [64.2–67.2] 39.0 [18.3–54.5] 26.0 [11.8–35.0] 11.5 [6.5–20.8] 5.0 [3.0-13.5] 2.5 [1.5–8.8] 1.5 [0.75-4.0]

Data are listed as median [IQR] or number (%). Abbreviations: ACE-I = Angiotensin converting enzyme inhibitor; ARB = angiotensin receptor blocker; COPD = Chronic obstructive pulmonary disease; GOLD = Global initiative for chronic obstructive lung disease; MLRD = mean lung radiation dose; MCRD = mean cardiac radiation dose; V = volume

**Fig. 1:**
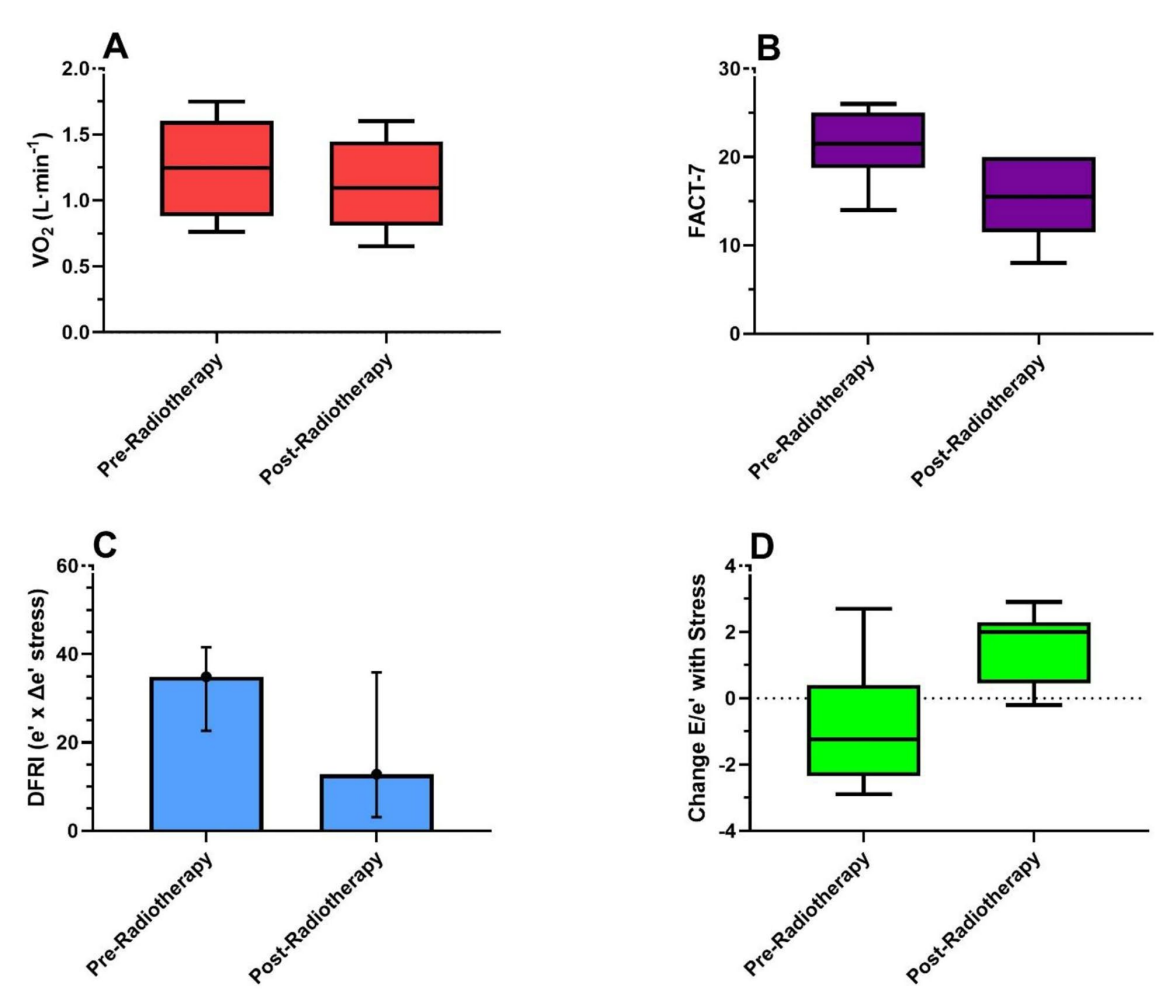
Pre- and post-radiotherapy box-whisker plots of significant variables **Panel A**: VO_2peak_. **Panel B**: FACT-G7 questionnaire scores. **Panel C**: Diastolic functional reserve index (DFRI). **Panel D**: Change in E/e’ with stress Abbreviations: VO_2_ = oxygen consumption; FACT-G7 = Functional Assessment of Cancer Therapy-General-7; DFRI = diastolic functional reserve index; E/e’= early transmitral flow [E] to early diastolic mitral annular velocity [e’] ratio

Table 2 displays the baseline and follow-up cardiopulmonary variables. Overall, the VO_2peak_ was impaired at baseline (70 [62–86] %-predicted) with 80% of patients having values < 85% of predicted consistent with impaired CRF. The VO_2peak_ significantly declined at post-radiotherapy follow-up assessments for absolute (Fig. 1), relative, and %-predicted VO_2peak_ expressions (*P* = 0.047; *P* = 0.047; *P* = 0.005), respectively. Furthermore, the proportion of patients that met criteria for functional disability was 7/10 (70%) and 8/10 (80%) at the baseline and follow-up assessments, respectively. A significant reduction in the OUES was observed between the baseline and follow-up assessments (1.63 [1.27–1.88] to 1.57 [1.12–1.75], *P* = 0.032). The OUES at baseline and follow-up demonstrated a strong positive correlation with VO_2peak_ (*R* = 0.818, *P* = 0.004 & *R* = 0.888, *P* < 0.001) reflecting its ability to track with VO_2peak_ in the scenario of a suboptimal exercise effort.

**Table 2:** Longitudinal Assessment of Cardiopulmonary Function in Lung Cancer Patients Undergoing Radiotherapy

**Body Composition & Physical Activity**			
	**Baseline**	**Follow-up**	***P*** **-value**
Body mass index, kg/m^2^	30.2 [24.4–35.1]	30.2 [23.4–35.2]	0.878
Physical Activity, MET/Min/week	1152 [122–1841]	759 [282–2804]	0.333
**Health-related Quality of Life**			
FACT-G7	21.5 [18.8–25.0]	15.5 [11.5–20.0]	**0.021**
**Lab Values**			
Hemoglobin, g/dL	12.7 [11.4–14.3]	12.6 [11.5–13.4]	0.102
hsCRP, mg/L	4.4 [1.4–5.8]	6.1 [3.7–20.7]	**0.047**
NT-proBNP, pg/mL	65 [49–125]	121 [88–191]	0.110
**Pulmonary Function**			
FVC, %	84 [62–98]	84 [66–98]	0.959
FEV-1, %	75 [60–102]	79 [65–102]	0.575
FEV-1/FVC ratio	0.71 [0.64–0.85]	0.75 [0.70–0.83]	0.553
DLCO-corrected, %	62 [51–80]	60 [59–67]	0.779
**Cardiopulmonary Exercise Test Variables**			
VO_2peak_, L·min^− 1^	1.245 [0.882–1.605]	1.095 [0.810–1.448]	**0.047**
VO_2peak_, %-predicted	70 [62–86]	62 [56–76]	**0.005**
VO_2peak_, mL·kg^− 1^·min^− 1^	13.8 [11.9–20.3]	13.5 [10.6–18.0]	**0.047**
VE/VCO_2_ slope	31.0 [27.5–35.1]	32.7 [29.1–38.9]	0.139
OUES	1.63 [1.27–1.88]	1.57 [1.12–1.75]	**0.032**
Peak RER	1.11 [1.04–1.14]	1.12 [1.04–1.20]	0.241
Breathing reserve, %	61 [51–85]	65 [49–73]	0.515
Exercise time, sec	418 [355–707]	405 [331–643]	0.169
Resting heart rate, bpm	82 [63–84]	83 [70–92]	0.838
Peak heart rate, bpm	127 [119–148]	124 [118–134]	0.241
Resting systolic blood pressure, mmHg	139 [126–149]	122 [111–133]	0.109
Resting diastolic blood pressure, mmHg	81 [76–83]	78 [70–83]	0.539
Peak systolic blood pressure, mmHg	176 [152–198]	163 [138–191]	0.114
Peak diastolic blood pressure, mmHg	78 [74–86]	71 [70–76]	**0.008**
**Rest Echo Parameters**			
Left-ventricular ejection fraction, %	62 [56–65]	66 [57–68]	0.475
Left-atrial volume index, mL/m^2^	26.9 [18.6–33.3]	24.3 [18.7–27.2]	0.646
E, cm/sec	71 [62–86]	72 [53–84]	0.285
A, cm/sec	81 [68–97]	86 [73–101]	0.093
E/A ratio	0.83 [0.69–1.11]	0.78 [0.66–0.96]	0.114
Septal e’, cm/sec	6.5 [5.5–8.2]	8.7 [7.7–11.1]	**0.028**
Lateral e’, cm/sec	9.8 [7.5–11.4]	11.2 [7.5–11.6]	0.646
Average e’, cm/sec	8.4 [6.5-9.0]	9.5 [7.5–10.7]	0.203
E/e’	7.9 [7.3–12.8]	7.5 [6.6–9.9]	0.169
**Stress Echo Parameters**			
E, cm/sec	105 [87–127]	104 [74–116]	0.508
Septal e’, cm/sec	11.0 [9.5–13.3]	10.4 [8.6–12.3]	0.173
Lateral e’, cm/sec	13.1 [11.9–17.0]	11.8 [8.6–15.1]	0.139
Average e’, cm/sec	12.9 [11.0-13.7]	11.3 [8.5–13.4]	0.126
Δstress e’, cm/sec	4.5 [3.4–5.2]	1.3 [0.3–4.5]	**0.022**
E/e’	8.3 [5.5–10.7]	8.4 [7.1–11.3]	0.386
DFRI (e’ x Δstress e’)	34.9 [22.7–41.6]	12.8 [3.1–35.9]	**0.037**

Data are listed as median [IQR] or number (%). **Bold** values = *P* < 0.05 for comparison between baseline and follow-up visit

Abbreviations: Δ = delta; A = late transmitral velocity; DFRI = diastolic functional reserve index; DLCO = diffusion capacity of carbon monoxide; E = early transmitral velocity; e’= early diastolic mitral annular velocity; FACT-G7 = functional assessment of cancer therapy-general-7; FEV-1 = forced expiratory volume at 1-second; FVC = forced vital capacity; hsCRP = high-sensitivity C-reactive protein; MET = metabolic equivalent of task; NT-proBNP = N-terminal pro-brain natriuretic peptide; OUES = oxygen uptake efficiency slope; RER = respiratory exchange ratio; VE/VCO_2_ = minute ventilation to carbon dioxide production; VO_2_ = oxygen consumption

We found a significant decline in the DFRI (34.9 [22.7–41.6] vs. 12.8 [3.1–35.9], *P* = 0.037) that was driven by an attenuated interval change in e’ after stress (4.5 [3.4–5.2] vs. 1.3 [0.3–4.5] cm/sec, *P* = 0.022; Fig. 1). Similarly, the change in Doppler E/e’ after stress (*inverse* measure of diastolic reserve) was significantly increased (-1.2 [-2.4, + 0.4] to + 2.0 [+ 0.4, + 2.3], *P* = 0.028; Fig. 1). Pre-treatment echocardiography revealed a LVEF of 62 [56–65] % with no significant change post-radiotherapy (66 [57–68] %).

The FACT-G7 score significantly decreased from 21.5 [18.8–25] to 15.5 [11.5–20] (*P* = 0.021) post-radiotherapy reflecting an interval decline in HRQOL. The hsCRP was above normal (≥ 1 mg/L) at 4.4 [1.4–5.8] in 9/10 (90%) of the subjects at baseline and increased significantly post-radiotherapy to 6.1 [3.7–20.7] mg/L, *P* = 0.047 with 9/10 (90%) of the subjects having elevated hsCRP levels at post-radiotherapy assessment. There was a trend toward increased levels of NT-proBNP (65 [49–125] to 121 [88–191] pg/mL, *P* = 0.110) at the post-radiotherapy assessment with 2/10 (20%) subjects having elevated NT-proBNP levels (≥ 125 pg/mL) at baseline and 4/10 (40%) having elevated NT-proBNP values post-radiotherapy. No significant interval changes were noted in BMI, physical activity levels, hemoglobin, pulmonary function (FVC, FEV-1, DLCO), or rest/exercise heart rates or blood pressures at post-radiotherapy follow-up assessments.

## Discussion

Cardiopulmonary exercise testing is the gold standard for evaluating integrative cardiovascular function and provides the unique advantage of objective quantifiable measures of CRF that are independent predictors of lung cancer morbidity, mortality, and overall quality of life [10, 39, 40]. This longitudinal, multidisciplinary study coupling functional cardiopulmonary and cardiac imaging studies demonstrates significant early changes in CRF and diastolic reserve are evident in patients with lung cancer following radiotherapy. A 12%, 2%, and 11% decrease (absolute change: -8 [-5 to -12%]), respectively, was observed in absolute, relative, and percent-predicted VO_2peak_ values between baseline and follow-up assessments performed 6 months following completion of radiotherapy. Furthermore, no significant differences were found between pre- and post-radiotherapy changes in body habitus (BMI), physical activity participation (IPAQ), pulmonary function results (FVC, FEV-1, DLCO), or hemoglobin levels, all of which have the potential to influence the CRF response.

In a population of apparently healthy adults, Imboden et al. described longitudinal changes in directly-measured CRF adjusted for time (mean time of 8.6 years between CRF assessments), baseline VO_2peak_, age, sex, and traditional risk factors and demonstrated a 1 mL·kg^− 1^·min^− 1^ change in VO_2peak_ was inversely associated with a ∼ 11, 15, and 16% respective risk for all-cause, CVD, and cancer mortality [41]. Similarly, studies by Chiaranda et al., using a different expression of CRF demonstrated that each 1% unit change in percent-predicted VO_2peak_ was associated with a 3% hospital admission and/or 3% mortality risk in patients with cardiovascular disease [42, 43]. These studies demonstrate that small longitudinal changes in VO_2peak_ can have significant impact.

In this study, we demonstrated interval declines in indices of diastolic reserve following radiotherapy that were concurrent with reductions in CRF. Our findings of declines in CRF, concurrent reductions in DFRI, and increases in the change in E/e’ after stress (*inverse* measure of diastolic reserve) reflects impaired myocardial relaxation or elevated filling pressures with exercise may be contributing to the reductions in CRF. This corroborates previous work in patients with lung cancer following radiotherapy without established CVD or heart failure that demonstrated reduced CRF is associated with measures of diastolic function (DFRI, E/e’) and biomarkers of ventricular wall stress [21, 24]. Although the general concept of RICD presenting as a predominantly diastolic dysfunction phenotype is widely accepted, there is surprisingly sparse literature to date incorporating measurements of diastolic function into the clinical assessment or study of patients following anti-cancer radiotherapy. Abnormal diastolic function impairs exercise capacity, which is likely a main contributor to the reduced HRQOL seen in patients with diastolic dysfunction. While studies have largely focused on diastolic dysfunction in patients with reduced ejection fraction, several studies have shown reductions in HRQOL in patients with diastolic dysfunction and preserved ejection fraction, often times being attributed to elevated filling pressures, which typically presents as dyspnea and fatigue [44, 45]. 

We also observed a significant increase in hsCRP post radiotherapy. Systemic inflammation following radiotherapy has been previously reported and associated with cardiac dysfunction [46–50]. 

### Study limitations

Limitations of this pilot study include the small sample size, single-site location, lack of a control group, confounding by the lung cancer disease process itself and potential contributions of the chemotherapeutic and immunotherapy agents, and lack of dedicated studies evaluating musculoskeletal function. Additionally, this study was limited to patients who completed both baseline and post-radiotherapy follow-up assessments, with a variable time to final assessment, and may be subject to selection/ recruitment bias. Strengths to this small study include the longitudinal study design and comprehensive assessment of non-cardiac causes of impaired CRF including respiratory function testing, hemoglobin status, and dedicated assessments of exercise diastolic function.

## Conclusions

Our preliminary findings indicate that a decline in cardiorespiratory fitness can be detected within the first six months following radiotherapy in patients with lung cancer and aligns with established research in the field describing declines in cardiorespiratory fitness that occur in patients with cancer undergoing cancer-related treatment. These data suggest that serial changes in cardiorespiratory fitness and cardiac diastolic reserve may serve as early markers to evaluate the potential effects of radiation therapy. However, these findings should be regarded as hypothesis-generating only and do not infer causality. Larger confirmatory studies aimed at addressing potential confounders as well as further investigation into the pathophysiology underlying the observed changes are warranted.

## Data Availability

The data analyzed during this study are available from the corresponding author upon reasonable request.

## Abbreviations

COPD: Chronic obstructive pulmonary disease
CPET: Cardiopulmonary exercise testing
CRF: Cardiorespiratory fitness
CVD: Cardiovascular disease
DFRI: Diastolic functional reserve index
DLCO: Diffusing capacity of lung for carbon monoxide
E/e: Early transmitral flow to early diastolic mitral annular velocity ratio
FACT-G7: Functional Assessment of Cancer Therapy-General-7
FEV-1: Forced expiratory volume-1second
FVC: Forced vital capacity
HRQOL: Health-related quality of life
hsCRP: High-sensitivity C-reactive protein
LVEF: Left ventricular ejection fraction
NT-proBNP: N-terminal pro-brain natriuretic peptide
OUES: Oxygen uptake efficiency slope
RICD: Radiation-induced cardiac disease
V: Volume
VO_2_: Oxygen consumption
